# Rapid and Sensitive Detection of *Vibrio* *parahaemolyticus* and *Vibrio vulnificus* by Multiple Endonuclease Restriction Real-Time Loop-Mediated Isothermal Amplification Technique

**DOI:** 10.3390/molecules21010111

**Published:** 2016-01-19

**Authors:** Yi Wang, Dongxun Li, Yan Wang, Kewei Li, Changyun Ye

**Affiliations:** 1 State Key Laboratory of Infectious Disease Prevention and Control, National Institute for Communicable Disease Control and Prevention, Collaborative Innovation Center for Diagnosis and Treatment of Infectious Diseases, Chinese Center for Disease Control and Prevention, Changping, Changbai Road 155, Beijing 102206, China; wildwolf0101@163.com (Y.W.); wangyan@icdc.cn (Y.W.); 2 Changping District Center for Disease Control and Prevention, Changping, Beijing 102200, China; xuner1989@163.com; 3 Institute of Microbiology, Jilin Provincial Center for Disease Control and Prevention, Changchun, Jilin 130062, China; likeweihaotingting@163.com

**Keywords:** *V*. *parahaemolyticus*, *V*. *vulnificus*, MERT-LAMP, LoD, LAMP

## Abstract

*Vibrio parahaemolyticus* and *Vibrio vulnificus* are two marine seafood-borne pathogens causing severe illnesses in humans and aquatic animals. In this study, a recently developed novel multiple endonuclease restriction real-time loop-mediated isothermal amplification technology (MERT-LAMP) were successfully developed and evaluated for simultaneous detection of *V*. *parahaemolyticus* and *V*. *vulnificus* strains in only a single reaction. Two MERT-LAMP primer sets were designed to specifically target *toxR* gene of *V*. *parahaemolyticus* and *rpoS* gene of *V*. *vulnificus*. The MERT-LAMP reactions were conducted at 62 °C, and the positive results were produced in as short as 19 min with the genomic DNA templates extracted from the *V*. *parahaemolyticus* and *V*. *vulnificus* strains. The two target pathogens present in the same sample could be simultaneously detected and correctly differentiated based on distinct fluorescence curves in a real-time format. The sensitivity of MERT-LAMP assay was 250 fg and 125 fg DNA per reaction with genomic templates of *V*. *parahaemolyticus* and *V*. *vulnificus* strains, which was in conformity with conventional LAMP detection. Compared with PCR-based techniques, the MERT-LAMP technology was 100- and 10-fold more sensitive than that of PCR and qPCR methods. Moreover, the limit of detection of MERT-LAMP approach for *V*. *parahaemolyticus* isolates and *V*. *vulnificus* isolates detection in artificially-contaminated oyster samples was 92 CFU and 83 CFU per reaction. In conclusion, the MERT-LAMP assay presented here was a rapid, specific, and sensitive tool for the detection of *V*. *parahaemolyticus* and *V*. *vulnificus*, and could be adopted for simultaneous screening of *V*. *parahaemolyticus* and *V*. *vulnificus* in a wide variety of samples.

## 1. Introduction

*Vibrio parahaemolyticus* and *Vibrio vulnificus*, which are the halophilic bacteria commonly found in the estuarine, coastal, and marine environments, belong to the family *Vibrionaceae* and cause seafood-borne gastrointestinal disorders in humans [1]. Ingestion of raw or undercooked seafood with the presence of *V. parahaemolyticus* and *V. vulnificus* is a risk factors in humans [2]. *V. parahaemolyticus* is considered as a major cause of seafood-caused diseases and has become a significant public health concern in many countries [3]. *V. vulnificus* is regarded as the leading cause of seafood-associated deaths in some countries, and responsible for over 95% of such fatal incidences in the United States [2]. Moreover, occasional exposure of open wounds to *V. parahaemolyticus* and *V. vulnificus* may initiate wound infection that may develop into septicemia in severe cases [4]. In this regard, rapid, specific, and reliable diagnostic techniques are particularly required to facilitate better detection and control of *V. parahaemolyticus* and *V. vulnificus* in seafood, environment, and clinical samples.

The traditional culture- and biochemical-based approaches for detection of *V. parahaemolyticus* and *V. vulnificus* involve enrichment in liquid media and subsequent isolation of colonies on selective media [5]. Although widely performed, such assays are laborious and time-consuming, requiring more than three days [5]. Additionally, several *Vibrio* species show similar biochemical characteristics that make it extremely difficult for rapid detection of *V. parahaemolyticus* and *V. vulnificus* [6]. Molecular-based techniques, such as PCR and real-time PCR assays, have been established for detection of *V. parahaemolyticus* and *V. vulnificus*, and produced rapid and specific results [7,8,9]. However, the PCR-based techniques rely on sophisticated apparatus or complex sample-handling procedures, and these requirements have been hampering its more flexible and wider applications [10,11]. Therefore, the establishment of alternative methodologies for simple, rapid, sensitive, and specific detection of *V. parahaemolyticus* and *V. vulnificus* is in continuous demand.

As a simple, rapid, and sensitive diagnosis technique, loop-mediated isothermal amplification (LAMP) has been applied to detect various pathogenic organisms, such as bacteria, fungi, virus, and parasites [12,13]. Up to now, the LAMP technique have been adopted for detecting *V. parahaemolyticus* and *V. vulnificus* in seafood and environment samples [14,15]. However, this amplification technology has been restricted to detect a single target, limiting the applicability of this assay [16,17]. A variety of studies have demonstrated strategies of multiplex LAMP (mLAMP) detection. However, they have been hampered in utility. Previous mLAMP assays have used end point analysis, through pyrosequencing or agarose gel techniques, while these approaches required further equipment or post amplification analysis, and did not allow real-time detection [18]. As such, a novel mLAMP methodology, which integrates real-time amplification and detection of multiple target sequences in a single reaction tube, will be valuable.

More recently, we have devised multiple endonuclease restriction real-time loop-mediated isothermal amplification technique (MERT-LAMP), which overcame the limitations posed by current LAMP technologies [19,20]. In the MERT-LAMP system, the new EFIP/EBIP primer was constructed, which was an extension of the forward/backward inner primer with an endonuclease recognition site at the 5′ end. EFIP/EBIP was modified at 5′ end with a fluorophore and in the middle with a dark quencher. One fluorophore was assigned to one target primer set and the appropriate quencher was matched to the fluorophore used. The restriction endonuclease Nb.*BsrDI* recognized and digested the newly synthesized double-stranded terminal sequences, which releases the quenching, resulting in a gain of sign. Thus, the MERT-LAMP approach allowed for real-time detection of multiple, distinct targets in an amplification reaction, avoiding post-reaction processing for analysis. Under isothermal conditions, MERT-LAMP reactions were conducted with multiple primer sets and targets in a single vessel, which enabled detection of minute amounts of nucleic acid in a short time without a thermocycling machinery. Herein, the novel MERT-LAMP technique is a good choice for diagnostic amplification in research on food hygiene, point-of-care testing, “on-site” screening, and more.

Here, we established a rapid, sensitive, and specific testing method based on the MERT-LAMP approach for simultaneous detection of *V. parahaemolyticus* and *V. vulnificus* by targeting *toxR* (GenBankID: L11929) and *rpoS* (GenBankID: AY187681) genes, respectively, and determined the sensitivity and specificity using pure cultures and spiked oyster samples.

## 2. Results

### 2.1. Detection of toxR- and rpoS-LAMP Products

**Figure 1:**
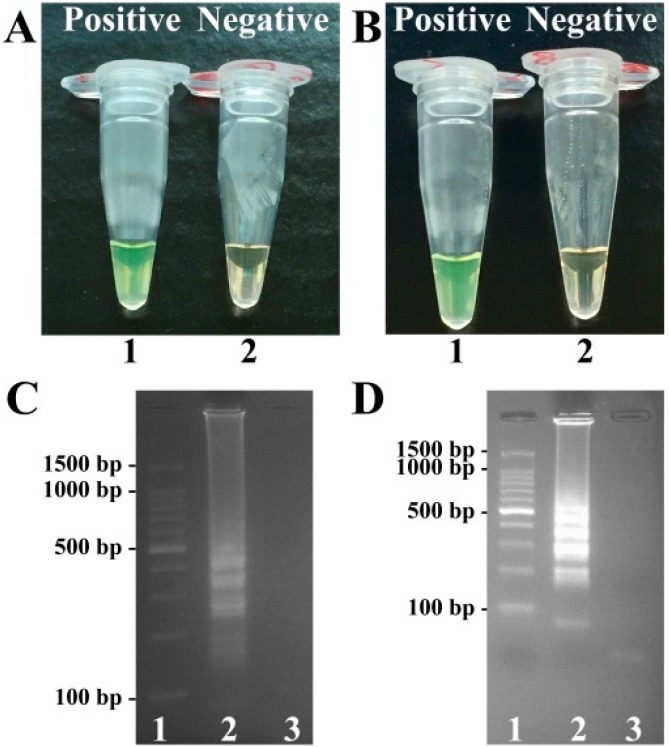
Confirmation and detection of *toxR*- and *rpoS*-LAMP products. (**A**,**B**), Color change of *toxR*- and *rpoS*-LAMP tubes; tube 1, positive reaction; tube 2, negative reaction; (**C**,**D**), 2% agarose gel electrophoresis applied to *toxR*- and *rpoS*-LAMP products; lane 1, DL 100-bp DNA marker; lane 2, positive LAMP reaction; lane 3, negative LAMP reaction.

To confirm the availability of *toxR-* and *rpoS*-LAMP primers, the LAMP reaction either for *V. parahaemolyticus* strains or *V. vulnificus* strains was processed in the absence or presence of genomic templates at 62 °C. The amplification products were analyzed by visual inspection using FD reagent, and the positive amplifications in *V. parahaemolyticus*- and *V. vulnificus*-LAMP tubes directly saw color change from light gray to green within 1h incubation periods (Figure 1). The conventional LAMP products were also analyzed by 2% agarose gel electrophoresis, and positive results showed the typical ladder-like patterns on gel electrophoresis (Figure 1). Thus, the *toxR-* and *rpoS*-LAMP primers for *V. parahaemolyticus* and *V. vulnificus* detection were found good candidates for developing the MERT-LAMP assays.

### 2.2. Sensitivity *of* V. parahaemolyticus- and V. vulnificus-LAMP Assay in Pure Culture

**Figure 2:**
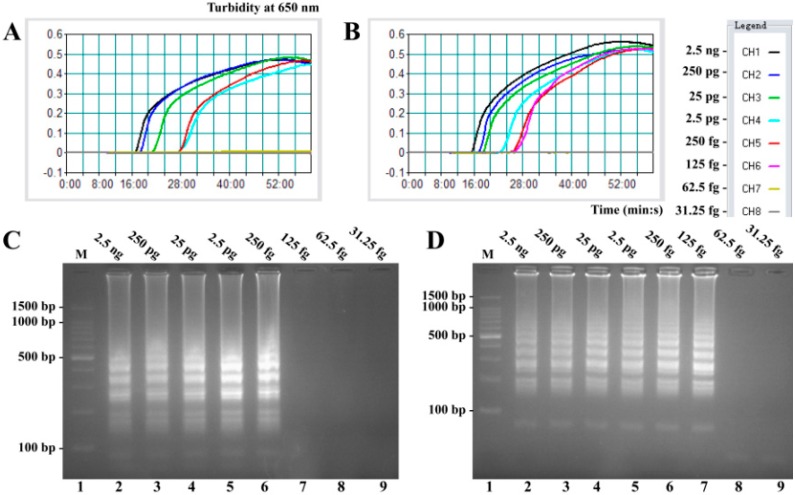
Sensitivity of the *toxR*- and *rpoS*-LAMP assays using serially-genomic DNA with *Vibrio parahaemolyticus* isolates and *Vibrio vulnificus* isolates as templates. LoD of *toxR*-LAMP (**A**) and *rpoS*-LAMP (**B**) for *V. parahaemolyticus* and *V. vulnificus* detection was monitored by real-time measurement of turbidity and the corresponding curves of concentrations of genomic DNA were marked in the figure. The sensitivity of *toxR*-LAMP assay was 250 fg per reaction, and the *rpoS*-LAMP for 125 fg per reaction. Sensitivity of *toxR*-LAMP (**C**) and *rpoS*-LAMP (**D**) for *V. parahaemolyticus* and *V. vulnificus* detection was analyzed by 2% agarose gel electrophoresis, and the positive amplifications were observed as a ladder-like pattern on 2% agarose gel electrophoresis analysis. Lane 1, DL 100-bp DNA marker.

Sensitivity of conventional LAMP assays on *V. parahaemolyticus* and *V. vulnificus* were evaluated by analyzing products produced from the serial dilutions (2.5 ng, 250 pg, 25 pg, 2.5 pg, 250 fg, 125 fg, 62.5 fg, and 31.25 fg per microliter) of the *V. parahaemolyticus* and *V. vulnificus* genomic DNA templates in triplicate. As shown in Figure 2, the LAMP reactions were analyzed by real-time turbidity detection; the limit of detection (LoD) of *V. parahaemolyticus*-LAMP assay was 250 fg per tube (Figure 2A), and the *V. vulnificus*-LAMP assay for 125 fg per tube (Figure 2B). Moreover, the final LAMP products were also detected by 2% agarose gel electrophoresis, and positive amplifications were observed as a ladder-like pattern (Figure 2C,D). The LoD of the agarose gel electrophoresis detection for *V. parahaemolyticus*- and *V. vulnificus*-LAMP reactions was in complete conformity with turbidity measurements.

### 2.3. Detection of MERT-LAMP Products in Non-Real-Time Format

**Figure 3:**
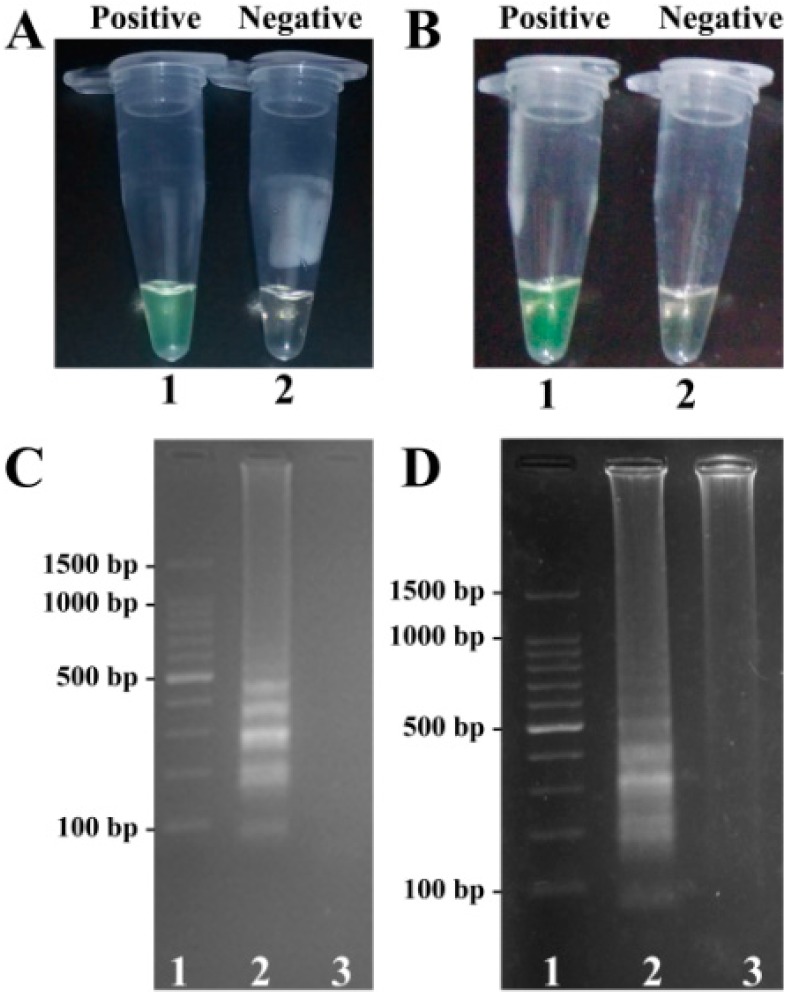
Confirmation and detection of toxR- and rpoS-MERT-LAMP products. (**A**,**B**), color change of *toxR*- and *rpoS*-MERT-LAMP tubes; tube 1, positive amplification; tube 2, negative amplification; (**C**,**D**), 2% agarose gel electrophoresis applied to *toxR*- and *rpoS*-MERT-LAMP products; lane 1, DL 100-bp DNA marker; lane 2, positive LAMP reaction; lane 3, negative LAMP reaction.

In order to demonstrate the utility of two MERT-LAMP primer sets, the MERT-LAMP assay either for *V. parahaemolyticus* strains or *V. vulnificus* strains was processed in the presence or absence of genomic DNA templates according to the standard MERT-LAMP condition. The MERT-LAMP products were detected by visual inspection using FD reagent, and the color change of positive MERT-LAMP reactions in *V. parahaemolyticus*- and *V. vulnificus* tubes from light gray to green were directly observed by naked eyes within 1 h incubation periods (Figure 3A). Furthermore, the MERT-LAMP products were also detected by 2% agarose gel electrophoresis, and the typical ladder-like patterns were observed but not in negative control (Figure 3).

### 2.4. The Optimal Amplification Temperature of MERT-LAMP Approach

**Figure 4:**
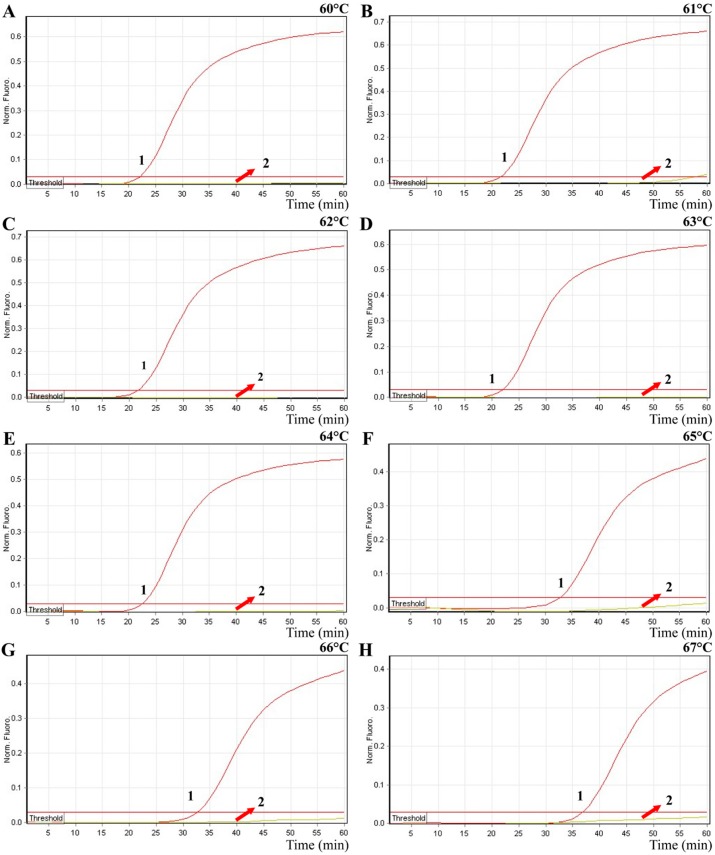
The optimal temperature for MERT-LAMP method. The MERT-LAMP amplifications were analyzed by means of real-time format, and the corresponding curves of DNA concentrations were listed. Signal 1 indicates *V. vulnificus* strains of ATCC 27562, and signal 2 indicates negative control. Eight kinetic graphs (**A**–**H**) were yielded at different amplification temperature (60–67 °C) with *V. vulnificus* genomic DNA at the level of 250 pg per tube. The graphs from 60 to 64 °C show robust amplification.

In order to test the optimal detection temperature, the MERT-LMAP amplifications were performed at distinct temperatures (60 to 67 °C) with *V. vulnificus*-MERT-LAMP primer set according to the standard MERT-LAMP reaction. The reference strain ATCC 27562 was selected as a positive control to examine the optimal temperature at the level of 250 pg of genomic DNA template per vessel. The results were detected by means of real-time format, and the typical kinetics graphs were yielded (Figure 4). Each amplification temperature produced a robust signal corresponding to Cy5 channel, with the faster amplifications were obtained from reaction temperatures of 60 to 64 °C, which were considered as the reference temperature for MERT-LAMP test. The detection temperature of 62 °C was applied for the rest of MERT-LAMP reactions conducted in this study.

### 2.5. Sensitivity of MERT-LAMP Assay for a Single Target

**Figure 5:**
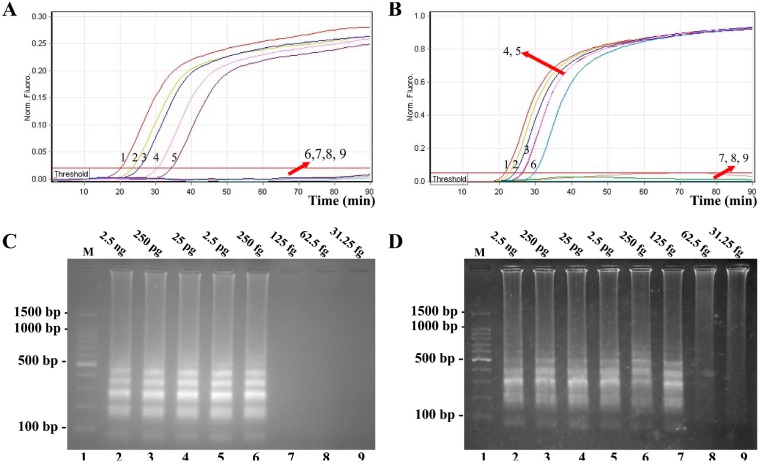
LoD of the *toxR*- and *rpoS*-MERT-LAMP approaches using serially-genomic DNA with *V. parahaemolyticus* strains and *V. vulnificus* strains as templates. Sensitivity of *toxR*-MERT-LAMP (**A**) and *rpoS*-MERT-LAMP (**B**) for *V. parahaemolyticus* and *V. vulnificus* detection was analyzed by real-time format, and signals 1, 2, 3, 4, 5, 6, 7, 8, and 9 represent DNA levels of 2.5 ng, 250 pg, 25 pg, 2.5 pg, 250 fg, 125 fg, 62.5 fg, and 31.25 fg per vessel and negative control. The LoD of *toxR*-MERT-LAMP assay was 250 fg per reaction, and the *rpoS*-MERT-LAMP for 125 fg per reaction. LoD of *toxR*-MERT-LAMP (**C**) and *rpoS*-MERT-LAMP (**D**) for *V. parahaemolyticus* and *V. vulnificus* detection was detected by 2% agarose gel electrophoresis, and the positive amplifications were seen as a ladder-like pattern on 2% agarose gel electrophoresis analysis. Lane 1, DL 100-bp DNA marker.

In the present study, we tested the MERT-LAMP approach in a single target format by using separate amplification of *toxR* (*V. parahaemolyticus*-specific gene) and *rpoS* (*V. vulnificus*-specific gene) from an *V. parahaemolyticus* and *V. vulnificus* DNA templates in each reaction, respectively. The Hex and Cy5 fluorophores, which were used for yellow and red channels, were assigned to *toxR*- and *rpoS*-MERT-LAMP primer sets, respectively. As shown in Figure 5, the release of quenching was seen as a robust increase of Cy5 and Hex signals, and positive amplifications were generated in approximately 19 min. The LoD of MERT-LAMP technique for independently analyzing *toxR* and *rpoS* genes was 250 fg and 125 fg of genomic DNA templates per tube, respectively (Figure 5). Moreover, the final MERT-LAMP products were also analyzed by 2% agarose gel electrophoresis; the typical ladder-like patterns were visible in positive amplifications but not in negative reactions and control. The LoD of the real-time detection for *V. parahaemolyticus*- and *V. vulnificus*-MERT-LAMP amplifications was completed accordance with agarose gel electrophoresis measurement.

The LoD of PCR, real-time PCR, conventional LAMP, and MERT-LAMP approaches on *V. parahaemolyticus* was 25 pg, 2.5 pg, 250 fg and 250 fg per reaction, and on *V. vulnificus* was 25 pg, 2.5 pg, 125 fg and 125 fg per reaction, respectively (Table 1, Figure 2 and Figure 5). These results suggested that the sensitivity of MERT-LAMP assay for detecting a single target was 10-fold and 100-fold more sensitive than that of qPCR and PCR assays, while was identical with conventional LAMP technique.

**Table 1:** The sensitivity and time for single MERT-LAMP technique targeting *toxR* and *ropS* genes compared with that of qPCR and PCR methods.

Approaches ^1^	Isothermal Amplification ^2^	Multiplex Detection	Regions Recognized	LoD for *V. parahaemolyticus*/*V.* *vulnificus*(No./Reaction) ^3^	Fastest Time (min)	LoD Time (min) ^4^
MERT-LAMP	+	+	8	250 fg/125fg	19	35
LAMP	+	–	8	250 fg/125 fg	19	35
qPCR	–	+	3	2.5 pg/2.5 pg	35	60
PCR	–	+	2	25pg/25pg	150	150

^1^ MERT-LMAP, multiple endonuclease restriction real-time loop-mediated isothermal amplification, qPCR, quantitative real-time PCR; ^2^ +, yes; –, no; ^3^ No., number; ^4^ LoD, limit of detection. The LoD values are the lowest gnomic DNA level that was positively amplified in triplicate. The positive amplifications of qPCR methods were generated as c_t_ values, which were converted to time for detection.

### 2.6. Sensitivity of MERT-LAMP Assay for Multiple Targets in a Reaction

**Figure 6:**
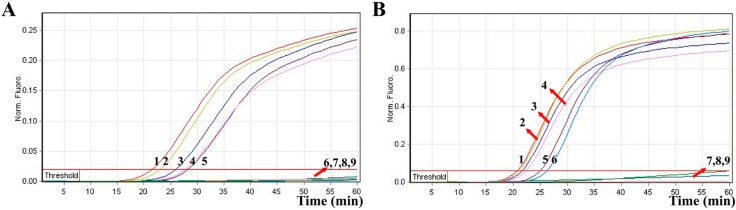
The LoD of multiplex MERT-LAMP method for simultaneously detecting two target pathogens. Two MERT-LMAP primer sets targeting *toxR* and *rpoS* genes were simultaneously added to a reaction vessel. (**A**,**B**) were simultaneously yielded from Hex (labeling *V. p*-EFIP of *toxR*) and Cy5 (labeling *V. v*-EFIP of *rpoS*) channels, respectively. LoD of multiplex-MERT-LAMP for simultaneously detecting *V. parahaemolyticus* (**A**) and *V. vulnificus* (**B**) was analyzed by real-time format, and signals 1, 2, 3, 4, 5, 6, 7, 8, and 9 represent DNA levels of 2.5 ng, 250 pg, 25 pg, 2.5 pg, 250 fg, 125 fg, 62.5 fg, and 31.25 fg per reaction and negative control. respectively. The sensitivity of multiplex MERT-LAMP approached for *V. parahaemolyticus* detection was 250 fg per tube, and the LoD of multiplex MERT-LAMP for *V. vulnificus* detection was125 fg per tube.

To examine the capability of MERT-LAMP technique for simultaneously detecting *V. parahaemolyticus* and *V. vulnificus* in a single reaction, we slightly adjusted the amount of the primers on the base of standard MERT-LAMP reaction, and the multiplex MERT-LAMP amplifications were carried out at 62 °C for 60 min. Two different fluorescence curves were simultaneously obtained from multiplex MERT-LAMP reactions, which contained two complete primer sets and their corresponding genomic templates (Figure 6). The MERT-LAMP methodology successfully detected *V. parahaemolyticus* and *V. vulnificus* in a single reaction, and provided a robust signal for each target. The LoD of MERT-LAMP assay for simultaneously detecting *toxR* and *rpoS* genes was 250 fg and 125 fg of each genomic DAN template per tube, respectively (Figure 6). No difference of sensitivity was obtained between detecting multiple targets and a single target in a MERT-LAMP reaction.

### 2.7. Analytical Specificity of the Multiplex MERT-LAMP Approach

**Figure 7:**
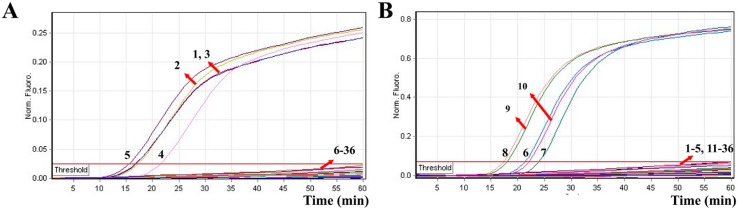
The specificity of multiplex MERT-LMAP technology for different strains. The multiplex MERT-LAMP reactions were carried out using different genomic DNA templates and were monitored by means of real-time format. (**A**,**B**) were simultaneously obtained from yellow (Hex) and red (Cy5) channels. Signals 1–5, *Vibrio parahaemolyticus* strains of ICDC-NVP001, ICDC-NVP002, ICDC-NVP003, ICDC-NVP004, ICDC-NVP005; signals 6–10, *Vibrio vulnificus* strains ATCC27562, ICDC-NVV001, ICDC-NVV002, ICDC-NVV003, ICDC-NVV004; signals 11–36, *Vibrio cholerae*, *Vibrio mimicus*, *Vibrio fluvialis*, *Vibrio algindyticus*, *Shigella flexneri*, *Shigella boydii*, *Shigella sonnel*, *Shigella* *dysenteriae*, *Salmonella choleraesuis*, *Listeria monocytogenes* (EGD-e), *Listeria monocytogenes* (ATCC19116), *Enterobacter cloacae*, *Enterococcus faecalis*, *Enteropathogenic E. coli*, *Enteroaggregative E. coli*, *Enterotoxigenic E. coli*, *Enteroinvasive E. coli*, *Enterohemorrhagic E. coli*, *Plesiomonas shigelloides*, *Yersinia enterocolitica*, *Cronobacter sakazakii*, *Staphylococcus aureus*, *Campylobacter jejuni*, *Pseudomonas aeruginosa* and *Bacillus cereus*, signal 36, negative control.

In order to test the MERT-LAMP technique’s specificity, the multiplex MERT-LAMP amplifications were conducted under the multiplex MERT-LAMP conditions described above with the purely genomic DNA templates extracted from 19 *V. parahaemolyticus*, 15 *V. vulnificus*, and 20 non-*V. parahaemolyticus* and non-*V. vulnificus* strains. By observation, positive results could be obtained only when genomic DNAs of *V. parahaemolyticus* strains and *V. vulnificus* strains were used as templates in multiplex MERT-LAMP reactions, and the target pathogens were correctly identified (Figure 7). However, for the reaction tubes of non-*V. parahaemolyticus* stains, non-*V. vulnificus* strains and negative control, there was no positive amplification after 60-min incubation period. These results demonstrated that the multiplex MERT-LAMP technology developed here was specific to target sequence identification.

### 2.8. Applicability of the MERT-LAMP Technology

**Figure 8:**
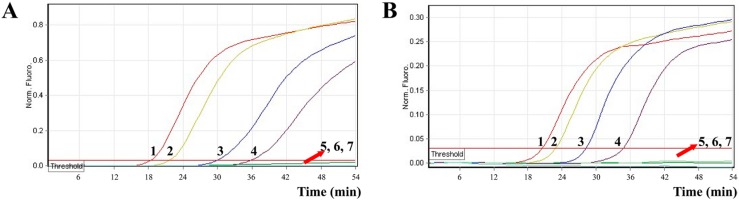
The LoD of MERT-LAMP approach for simultaneously detecting two target pathogens in artificially contaminated oysters samples. Two MERT-LMAP primer sets targeting *toxR* and *rpoS* genes were added to a reaction tube. (**A**,**B**) were simultaneously yielded from Hex (labeling *V. P*-EFIP of *toxR*) and Cy5 (labeling *V. v*-EFIP of *rpoS*) channels, respectively. LoD of MERT-LAMP for simultaneously detecting *V.* *parahaemolyticus* (**A**) and *V.* *vulnificus* (**B**) in artificially contaminated oyster samples were monitored by real-time format, and signals 1, 2, 3, 4, 5, 6, and 7 represent *V.* *parahaemolyticus* DNA levels of 9200 CFU, 920 CFU, 92 CFU, 9.2 CFU, 0.92 CFU, and 0.092 CFU per reaction and negative control; *V. vulnificus* DNA levels for 9300 CFU, 830 CFU, 83 CFU, 8.3 CFU, 0.83 CFU, and 0.083 CFU per reaction and negative control. The sensitivity of MERT-LAMP method for *V.* *parahaemolyticus* detection in artificially contaminated oyster samples was 92 CFU per reaction, and the LoD of MERT-LAMP for *V. vulnificus* detection in artificially contaminated oyster samples was 83 CFU per vessel.

In order to evaluate the practicability of MERT-LAMP approach as a surveillance tool for *V. parahaemolyticus* and *V. vulnificus* in seafood, the MERT-LAMP methodology was examined by artificially adding *V. parahaemolyticus* and *V. vulnificus* strains into oysters. The LoD of MERT-LAMP assay was 92 CFU/reaction of *V. parahaemolyticus* ICDC-NVP001 and 83 CFU/reaction of *V. vulnificus* ATCC 27562 in spiked oyster samples without enrichment, and the two target pathogens could be simultaneously detected in a single MERT-LAMP reaction (Figure 8, Table 2). The non-contaminated oyster sample was found to be negative.

The LoD of multiplex MERT-LAMP approach was in conformity with that of conventional LAMP assay only for *V. parahaemolyticus* or *V. vulnificus*, respectively (Table 2). Comparatively, the PCR and qPCR methods generated positive results when the contaminate numbers of *V. parahaemolyticus* ICDC-NVP001 amounted to more than 9200 and 920 CFU/reaction, *V. vulnificus* ATCC 27562 for 8300 and 830 CFU/reaction, respectively. The results suggested that the sensitivity of multiplex MERT-LAMP approaches was 100- and 10-fold more sensitive than that of PCR and qPCR technologies (Table 2).

**Table 2:** Comparison of MERT-LMAP, LAMP, qPCR, and PCR methods for detection of *V. parahaemolyticus* and *V.* *vulnificus.*in artificially contaminated oyster samples.

Detection Assays ^1^	Multiplex Detection ^2^	LoD (No./Reaction) ^3^	
		*V. parahaemolyticus* Detection	*V.* *vulnificus* Detection
MERT-LAMP	+	92 CFU	83 CFU
LAMP	–	92 CFU	83 CFU
qPCR	–	920 CFU	830 CFU
PCR	–	9200 CFU	8300 CFU

**^1^** MERT-LMAP, multiple endonuclease restriction real-time loop-mediated isothermal amplification; LAMP, loop-mediated isothermal amplification. ^2^ +, yes; –, no; ^3^ No., number.

## 3. Discussion

*V. parahaemolyticus* and *V. vulnificus*, as serious bacterial pathogens for aquatic animals and humans, are widely distributed in coastal and estuarine environments throughout the world [15]. In aquatic animals, the two pathogens are capable of causing serve illnesses in penaeid shrimp, fish, and shellfish, leading to significant losses in aquaculture industries [21]. In humans, most *V. parahaemolyticus* and *V. vulnificus* infections resulted from the consumption of raw, inadequately or improperly cooked seafood, particularly shellfish (such as oysters) [1]. Therefore, there is a need for rapid, specific, sensitive, and cost-effective diagnosis methodology that can be used effectively for simultaneous detection of *V. parahaemolyticus* and *V. vulnificus* in various samples.

In this report, the first MERT-LAMP assay targeting the *toxR* gene of *V. parahaemolyticus* and the *rpoS* gene of *V. vulnificus* has was successfully established. The new MERT-LAMP technique could simultaneously detect and distinguish *toxR* and *rpoS* genes in only one isothermal step, eliminating the use of temperature-regulating apparatus. Moreover, the MERT-LAMP did not require further processing, and the reaction tubes were not opened in the course of the experiment, which effectively alleviated any carryover contamination. The optimal MERT-LAMP amplification temperature was 60 °C to 64 °C, and 62 °C was selected as reaction temperature. The MERT-LAMP reactions were completed within 60 min. The detection speed of MERT-LAMP method was similar with traditional LAMP assays, while the MERT-LAMP methodology achieved multiplex detection in a single reaction; thus, *V. parahaemolyticus* and *V. vulnificus* strains in the same samples could be simultaneously detected and correctly identified (Figure 6 and Figure 8).

Sensitivity of the MERT-LAMP assay was examined using serial dilution of genomic DNA templates. First, we evaluated the MERT-LAMP technique in a single target format by using separate detection of *toxR* (*V. parahaemolyticus*-specific gene) and *rpoS* (*V. vulnificus*-specific gene) from an *V. parahaemolyticus* and *V. vulnificus* DNA templates in each reaction, and the LoD of MERT-LAMP methodology for independently amplifying *toxR* gene or *rpoS* gene was 250 fg or 125 fg of genomic DNA templates per reaction, respectively (Figure 5). The sensitivity of MERT-LAMP method for detecting a single target was consistent with conventional LAMP assay (Figure 2 and Figure 5, Table 1). Second, we extended the MERT-LAMP approach to simultaneously amplify *toxR* and *rpoS* genes in a single reaction, and the two target pathogens were successfully identified and detected. The LoD of MERT-LAMP approach for simultaneously detecting *toxR* and *rpoS* genes was 250 fg and 125 fg of genomic DNA templates per tube, respectively, and which is the same as that of singleplex MERT-LAMP detections (Figure 5 and Figure 6). The LoD of multiplex MERT-LAMP technology was identical with that the conventional LAMP technique described in this study or developed in other publications, and 10-fold and 100-fold more sensitive than conventional qPCR and PCR techniques (Figure 2 and Figure 6, Table 1) [19]. In addition, the multiplex MERT-LAMP assay was also successfully examined using artificially contaminated oyster samples, and the multiplex MERT-LAMP methodology performed better than PCR and qPCR techniques with respect to detection speed and LoD (Figure 8 and Table 2).

In addition to its sufficient sensitivity, the MERT-LAMP assay in identification of *V. parahaemolyticus* and *V. vulnificus* offered a high degree of specificity and the assay specificity was successfully ascertained. Each primer set contained six specific primers that recognized eight distinct regions on the *toxR* gene of *V. parahaemolyticus* and *rpoS* gene of *V. vulnificus*, which ensured high specificity of the DNA template detection [7,9,19]. In order to verify the specificity of multiplex MERT-LAMP for detection of *V. parahaemolyticus* and *V. vulnificus*, a variety of bacterial strains listed in Table 3 were determined in this study. The results obtained from our study showed that the MERT-LAMP technology indicated a high degree of specificity to *V. parahaemolyticus* and *V. vulnificus* by generating positive results to all examined isolates of the two bacteria while producing a negative result to non-*V. parahaemolyticus* and non-*V. vulnificus* bacteria strains. Moreover, the two target pathogens were correctly differentiated (Figure 7). These results suggested that the MERT-LAMP assay established here was specific to target sequence detection.

In conclusion, our study is the first report of the use of MERT-LAMP assay for simultaneous detection of *V. parahaemolyticus* and *V. vulnificus*, comparing with regular LAMP detection approaches, which provides advantages on real time and multiplex detection. Compared with PCR-based techniques, the MERT-LAMP method is advantageous on sensitivity, specificity, rapidity, time consumption, and ease in operation. Therefore, the developed MERT-LAMP assay can be used as a valuable tool for detection of *V. parahaemolyticus* and *V. vulnificus* in basic and field laboratories.

## 4. Materials and Methods

### 4.1. Design of the MERT-LAMP Primers

**Figure 9:**
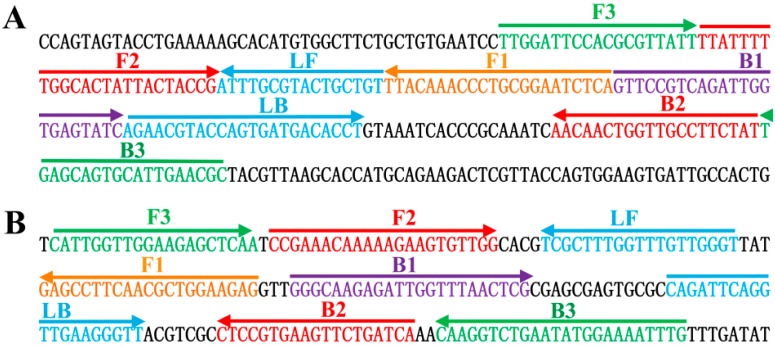
Location and sequence of *toxR* (*Vibrio parahaemolyticus*) and *rpoS* (*Vibrio vulnificus*) genes used to design MERT-LAMP primers. The nucleotide sequences of the sense strands of *toxR* (**A**) and *rpoS* (**B**) are showed. The sites of primer sequences were underlined. Right arrows and left arrows showed sense and complementary sequences that are used.

Based on the *toxR* gene of *V. parahaemolyticus* and *rpoS* gene of *V. vulnificus*, two sets of MERT-LAMP primers were designed by PrimerExplorer V4 (Eiken Chemical) according to the mechanism of MERT-LAMP methodology [7,9]. Blast analysis validated that two sets of MERT-LAMP primers were specific for *V. parahaemolyticus* and *V. vulnificus*. The dark quenchers used were Black Hole Quencher-1 and Black Hole Quencher-2, and the fluorophores used were HEX and Cy5, which are assigned to *toxR* and *rpoS* primer sets, respectively. All oligonucleotides described here were synthesized and purified by Tianyi Biotech (Beijing, China). The details of target sequences, primer design, primers sequences and locations were shown in Table 3 and Figure 9.

**Table 3:** The primers used in this study.

Primers Name ^1^	Sequences and Modifications	Length	Genes
*V.p*-F3	5′-TTGGATTCCACGCGTTATT-3′	19 nt	*toxR*
*V.p*-B3	5′-GCGTTCAATGCACTGCTCA-3′	19 nt	
*V.p*-FIP	5′-TGAGATTCCGCAGGGTTTGTAATTATTTTTGGCACTATTACTACCG-3′	46 mer	
*V.p*-BIP	5′-GTTCCGTCAGATTGGTGAGTATCATAGAAGGCAACCAGTTGTT-3′	43 mer	
*V.p*-EFIP	5′-HEX-TGCAATG-TGAGAT(BHQ1)TCCGCAGGGTTTGTAATTATTTTTGGCACTATTACTACCG-3′	53 mer	
*V.p*-LF	5′-ACAGCAGTACGCAAAT-3′	16 nt	
*V.p*-LB	5′-AGAACGTACCAGTGATGACACCT-3′	23 nt	
*V.v*-F3	5′-CATTGGTTGGAAGAGCTCAA-3′	20 nt	*rpoS*
*V.v*-B3	5′-CAAATTTTCCATATTCAGACCTTG-3′	24 nt	
*V.v*-FIP	5′-CTCTTCCAGCGTTGAAGGCTCTTTTCCGAAACAAAAAGAAGTGTTGG-3′	47 mer	
*V.v*-EFIP	5′-Cy5-TGCAATG-CTCT(BHQ-2)TCCAGCGTTGAAGGCTCTTTTCCGAAACAAAAAGAAGTGTTGG-3′	54 mer	
*V.v*-BIP	5′-GGGCAAGAGATTGGTTTAACTCGTTTTTGATCAGAACTTCACGGAG-3′	46 mer	
*V.v*-LF	5′-ACCCAACAAACCAAAGCGA-3′	19 nt	
*V.v*-LB	5′-CAGATTCAGGTTGAAGGGTT-3′	20 nt	

^1^ *V. p*, *Vibrio parahaemolyticus*; *V. v*, *Vibrio vulnificus*; F, forward; B, backward; FIP, forward inner primer; EFIP, the novel forward inner primer; BIP, backward inner primer; LF, loop forward primer; LB, loop backward primer.

### 4.2. Reagents

Loopamp^TM^ Fluorescent Detection Reagent (FD) and the Loopamp kits were purchase from Eiken Chemical (Tokyo, Japan, and Beijing, China). The DNA extraction kits (QIAamp DNA Mini Kits) were purchased from Qiagen (Beijing, China), and the Nb.*BsrDI* was purchased from New England Biolabs (Beijing, China).

### 4.3. Bacterial Strains

The bacterial strains tested in this study were listed in Table 4. All *Vibrio* strains were routinely cultured using thiosulfate citrate bile salt sucrose agar (TCBS agar, Eiken Chemical) at 35 °C overnight. Storage and culture conditions of non-*Vibrio* strains were followed as previously described [11].

**Table 4:** Bacterial strains used in this study.

Bacteria	Strain No. (Source of Strain) ^1^	No. of Strains
*Vibrio parahaemolyticus*	ICDC-NVP001	1
	Isolated strains	26
*Vibrio vulnificus*	ATCC27562	1
	Isolated strains	11
*Vibrio cholerae*	ATCC14035	1
	Isolated strains	1
*Vibrio mimicus*	Isolated strains	1
*Vibrio fluvialis*	Isolated strains	1
*Vibrio alginolyticus*	Isolated strains	1
*Enterohemorrhagic E. coli*	EDL933	1
*Enteropathogenic E. coli*	Isolated strains	1
*Enterotoxigenic E. coli*	Isolated strains	1
*Enteroaggregative E. coli*	Isolated strains	1
*Enteroinvasive E. coli*	Isolated strains	1
*Yersinia enterocolitica*	ATCC23715	1
*Shigella dysenteriae*	Isolated strains	1
*Shigella boydii*	Isolated strains	1
*Shigella flexneri*	Isolated strains	10
*Shigella sonnei*	Isolated strains	1
*Salmonella*	Isolated strains	10
*Plesiomonas shigelloides*	ATCC51903	1
*Listeria monocytogenes*	EGD-e	1
	ATCC19116	1
*Listeria ivanovii*	ATCCBAA-678	1
*Listeria grayi*	ATCC25402	1
*Enterococcus faecalis*	ATCC35667	1
*Enterobacter cloacae*	Isolated strains	1
*Cronobacter sakazakii*	Isolated strains	1
*Bacillus cereus*	Isolated strains	5
*Campylobacter jejuni*	ATCC33291	1
*Pseudomonas aeruginosa*	Isolated strains	10
*Staphylococcus aureus*	Isolated strains	10
*Klebsiella pneumoniae*	Isolated strains	5

**^1^** ATCC, American Type Culture Collection; ICDC, National Institute for Communicable Disease Control Disease Control and Prevention, Chinese Center for Disease Control and Prevention.

### 4.4. DNA Templates Preparation

According to the manufacturer’s instructions, DNA templates used in this study were extracted from all culture strains using DNA extraction kits (QIAamp DNA minikits; Qiagen, Hilden, Germany). The extracted templates were tested with ultraviolet spectrophotometer at A260/280 and stored under at −20 °C until use.

### 4.5. The Conventional LAMP Assay

In order to examine the availability of two LAMP primer sets, the LAMP amplification either for *V. parahaemolyticus* strains or *V. vulnificus* strains was carried out as the following description. Briefly, the traditional LAMP reaction was performed with the Loopamp Kit in a final volume of 25 μL containing 1.6 μM each FIP and BIP primers, 0.8 μM each LF and LB primers, 0.4 μM each F3 and B3 primers, 12.5 μL 2× reaction mix, 1 μL of *Bst* DNA polymerase (8 U), 1 μL FD and 1 μL DNA template. 

The amplification mixtures of convention LAMP were heated at 62 °C for 1 h then at 85 °C for 5 min to stop the amplification. Three methods were employed to monitor the conventional LAMP reaction. First, a real-time turbidimeter (LA-320C, Eiken Chemical Co., Ltd., Tokyo, Japan) was used to minor the LAMP reaction by recording the optical density (OD) at 650 nm every 6 s. The turbidity readings were produced in real-time format and a turbidity threshold value of 0.1 was defined. Second, the positive reactions could be directly seen color change by FD reagent. Moreover, the amplification products were also detected by electrophoresis on 2% agarose gels with ethidium bromide staining. Mixture without DNA template was used as a negative control.

### 4.6. The Standard MERT-LAMP Assay

In order to test utility of two MERT-LAMP primer sets, the reaction mixtures of MERT-LAMP were performed in a final volume of 25 μL containing 0.8 μM EFIP and FIP primers, 1.6 μM BIP primers, 0.8 μM each LF and LB primers, 0.4 μM each F3 and B3 primers, 12.5 μL 2× reaction mix, 1 μL (8 U) of *Bst* DNA polymerase, 1.5 μL (15 U) of Nb.*BsrDI* endonuclease and 1μL DNA template.

A real-time machine (Rotor-Gene Q, Qiagen, Hilden, Germany) was applied to run the MERT-LAMP reactions, and the assay was conducted at 62 °C for 1 h. Mixture without DNA template was used as a negative control. After amplification, the MERT-LAMP products were confirmed by electrophoresis on 2% agarose gels with ethidium bromide staining or directly observed the color change by FD reagent.

To assess the optimal reaction temperature of MERT-LAMP approach, we mixed the MERT-LAMP reaction mixtures at a constant temperature ranging from 60 °C to 67 °C for 60 min and then heated at 85 °C for 5 min to complete the reaction. Mixtures without DNA template were used as a negative control.

### 4.7. The Multiplex MERT-LAMP Reaction

For multiplex amplifications, the MERT-LAMP assay was performed as the following system: 25 μL containing 0.4 μM*V.v*-EFIP and *V.v*-FIP primers, 0.8 μM*V.v*-BIP primers, 0.4 μM each *V.v*-LF and *V.v*-LB primers, 0.4 μM each *V.v*-F3 and *V.v*-B3 primers,1.2 μM*V.p*-EFIP and *V.p*-FIP primers, 2.4 μM*V.v*-BIP primers, 1.2 μM each *V.v*-LF and *V.v*-LB primers, 0.4 μM each *V.v*-F3 and *V.v*-B3 primers, 12.5 μL 2× reaction mix, 1.5 μL (15 U) of Nb.*BsrDI* endonuclease,1 μL (8 U) of *Bst* DNA polymerase and 1 μL DNA template DNA each of *V. parahaemolyticus* strains and *V. vulnificus* strains. The MERT-LAMP reactions were conducted at 62 °C for 1 h in the real-time system, and amplification mixtures without the DNA template were used as a negative control. The lowest detectable template amount were determined in triplicate.

### 4.8. Determination of the Sensitivity of the MERT-LAMP in Pure Cultures

Strains of *V. parahaemolyticus* ICDC-NVP001 and *V. vulnificus* ATCC 27562 were used for the assay of sensitivity testing with pure culture, and the genomic DNA templates were serially diluted. Individual MERT-LAMP approaches, which target the *toxR* and *rpoS* genes individually, were carried out with serial dilutions (2.5 ng, 250 pg, 25 pg, 2.5 pg, 250 fg, 125 fg, 62.5 fg, and 31.25 fg per microliter) according to standard MERT-LAMP system. To make a comparative analysis of MERT-LAMP, LAMP, qPCR and PCR assays by using pure culture, the limit of detection (LoD) of LAMP, qPCR, and PCR assays was defined by genomic DNA amount of the template. The *V. parahaemolyticus*-qPCR, *V. parahaemolyticus*-PCR, *V. vulnificus*-qPCR, and *V. vulnificus*-PCR assays have been established in previous studies, which were selected for confirming the LoD of qPCR and PCR technologies [22,23,24,25].

To test the sensitivity of multiplex MERT-LAMP assay, the MERT-LAMP reaction was conducted under the conditions described above with serial dilutions (2.5 ng, 250 pg, 25 pg, 2.5 pg, 250 fg, 125 fg, 62.5 fg, and 31.25 fg per microliter) of *V. parahaemolyticus* and *V. vulnificus*, and 1μl DNA template DNA each of *V. parahaemolyticus* strains and *V. vulnificus* strains was simultaneously added into a MERT-LMAP reaction.

### 4.9. Evaluation of the Specificity of the MERT-LAMP Assay

To assess the MERT-LAMP technology’s specificity, the multiplex MERT-LAMP reactions were carried out under the conditions described above with the purely genomic DNA templates extracted from 111 strains (Table 2). Analysis of each sample was performed twice independently.

### 4.10. Practical Application of MERT-LAMP to V. parahaemolyticus and V. vulnificus Detection in Oyster Samples

In order to determine practicability of MERT-LAMP approach in identification of *V. parahaemolyticus* and *V. vulnificus*, *V. parahaemolyticus* (ICDC-NVP001), and *V. vulnificus* (ATCC27562) were simultaneously added to oyster samples, which were purchased at a local market in Beijing. The oyster samples were confirmed as being *V. parahaemolyticus* and *V. vulnificus* negative by traditional culture assay and PCR [23,25]. Only oyster samples, which were negative for *V. parahaemolyticus* and *V. vulnificus*, were applied to spiked oyster samples.

Firstly, to test the minimal detectable colony forming units (CFUs), the cultures with *V. parahaemolyticus* and *V. vulnificus* strains were serially diluted (10^−1^ to 10^−9^), and the aliquots of 100 μL appropriate dilution (10^−6^) was plated in triplicate on brain heart infusion (BHI). The CFUs were counted after 24 h at 37 °C [20]. The following procedures, one hundred microliters of appropriate dilutions of *V. parahaemolyticus* and *V. vulnificus* with known amounts was simultaneously spiked into 800 μL of each of the oyster homogenates and mixed well. The oyster homogenates was centrifuged at 200 g for 5 min and the supernatant was transferred to a new vessel, and then was centrifuged at 18,000 g for 5 min. After removal of the supernatant, the pellet was applied for DNA extraction. For MERT-LAMP, LAMP, real-time PCR, and PCR assays, 1 μL of each supernatants (2 μL) were used as templates. Non-contaminated oyster sample was chosen as negative control. This performance was carried out in triplicate independently.
